# Combined short- and long-axis ultrasound-guided central venous catheterization is superior to conventional techniques: A cross-over randomized controlled manikin trial

**DOI:** 10.1371/journal.pone.0189258

**Published:** 2017-12-07

**Authors:** Jun Takeshita, Kei Nishiyama, Satoru Beppu, Nozomu Sasahashi, Nobuaki Shime

**Affiliations:** 1 Department of Emergency and Critical Care Medicine, National Hospital Organization, Kyoto Medical Center, Kyoto, Japan; 2 Department of Emergency and Critical Care Medicine, Institute of Biomedical & Health Sciences, Hiroshima University, Hiroshima, Japan; Cardiff University, UNITED KINGDOM

## Abstract

**Objectives:**

Visualizing the needle tip using the short-axis (SA) ultrasound-guided central venous catheterization approach can be challenging. It has been suggested to start the process with the SA approach and then switch to the long-axis (LA); however, to our knowledge, this combination has not been evaluated. We compared the combined short- and long-axis (SLA) approach with the SA approach in a manikin study.

**Methods:**

We performed a prospective randomized controlled cross-over study in an urban emergency department and intensive care unit. Resident physicians in post-graduate years 1–2 performed a simulated ultrasound-guided internal jugular vein puncture using the SA and SLA approaches on manikins. Twenty resident physicians were randomly assigned to two equal groups: (1) one group performed punctures using the SA approach followed by SLA; and (2) the other performed the same procedures in the opposite order. We compared the success rate and procedure duration for the two approaches. Procedural success was defined as insertion of the guide-wire into the vein while visualizing the needle tip at the time of anterior wall puncture, without penetrating the posterior wall.

**Results:**

Six resident physicians (30%) performed both approaches successfully, while 12 (60%) performed the SLA approach, but not the SA, successfully. Those who performed the SA approach successfully also succeeded with the SLA approach. Two resident physicians (10%) failed to perform both approaches. The SLA approach had a significantly higher success rate than the SA approach (P < 0.001). The median (interquartile range) procedure duration was 59.5 [46.0–88.5] seconds and 45.0 [37.5–84.0] seconds for the SLA and SA approaches, respectively. The difference of the duration between the two procedures was 15.5 [0–28.5] seconds. There was no significant difference in duration between the two approaches (P = 0.12).

**Conclusions:**

Using the SLA approach significantly improved the success rate of internal jugular vein puncture performed by novice physicians on a manikin model, without increasing procedural duration. Further clinical trials are warranted to confirm the procedure’s utility in actual patients.

**Trial registration:**

UMIN Clinical Trials Registry UMIN000026199

## Introduction

Ultrasound-guided central venous catheterization has become common. Its success rate has significantly increased, while complications have decreased, compared with the conventional anatomical landmark-guided method [1–11].

There are two ultrasound-guided puncturing techniques: the short-axis (SA) and the long-axis (LA) approaches. In the former, the SA image of the target vein is acquired, and the vein is punctured perpendicularly to the ultrasound probe. The needle tip is then visualized as a dot on the screen of the ultrasonography monitor. The position of the target vein relative to the direction of needle advancement can be visualized, as can the adjacent vessels, such as the common carotid and vertebral arteries. However, it is sometimes difficult to visualize the needle tip during advancement when using the SA approach. This can result in posterior wall puncturing (PWP) of the target vein [12–14] or the inadvertent puncture of the common carotid artery and pneumothorax [15–17]. Alternatively, the needle tip is continuously visualized as it advances from the skin to the target veins when using the LA approach. However, it is technically difficult to detect the center of the target vein and visualize the entire length of the needle when using this approach. Discerning the position of the target vein in relation to adjacent vessels is also challenging when using this approach [18, 19], and the risk of arterial puncture increases. To facilitate the visualization of the needle in the LA view, it has been suggested that the procedure should begin using the SA approach, followed by the LA approach [20]. However, to our knowledge, no studies have demonstrated the utility of this combined technique.

We hypothesized that a combined short- and long-axis (SLA) approach, which would incorporate the advantages of the SA and LA approaches, would facilitate visualization of the needle tip and prevent PWP. The aim of this study was to compare the SLA approach with the SA approach, when administered by novice operators in a simulated model.

## Methods

### Ethical considerations

The study protocol conformed to the Guidelines for Epidemiologic Studies issued by the Ministry of Health, Labor, and Welfare of Japan, and was approved by the institutional review board of Kyoto Medical Center. Our work complies with the principles of the Declaration of Helsinki. This manuscript adheres to the applicable Equator guidelines.

### Study design and setting

**Fig 1:**
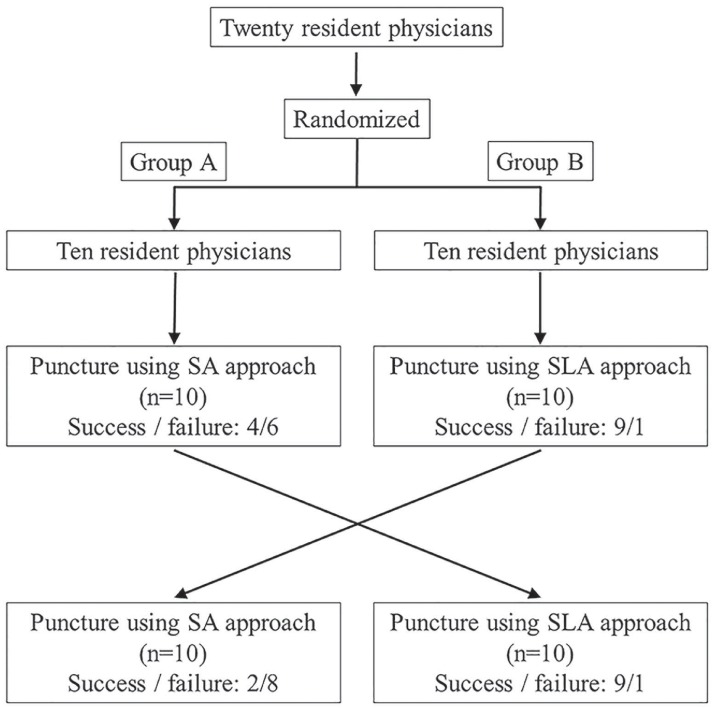
Study flow and outcomes. SA, short-axis; SLA, combined short- and long-axis.

This was a prospective randomized controlled cross-over study, performed in an urban emergency department and intensive care unit. The participants were residents in either their 1st or 2nd year post graduation from medical school. None of the residents had specialized in any particular branch of medicine at the time and all had very limited experience with central venous punctures in clinical practice (median: 0.5; interquartile range: 0–4.5 times). They were blinded to the study and were assigned to two groups using simple randomization without concealment and blinding. One of the investigators (J. T.) generated the random allocation sequence, enrolled participants, and assigned participants to the interventions. Puncturing was performed using a simulator model doll (M93B CVC Insertion Simulator II, Kyoto Kagaku Co., Ltd, Kyoto, Japan). Group A physicians first performed the SA approach followed by the SLA approach. Group B physicians performed the same procedures in the opposite order (Fig 1). No participants dropped out during recruitment and follow-up.

### Puncturing procedures

We used a Sonosite M-turbo Ultrasound System (Fujifilm SonoSite Japan, Inc., Tokyo, Japan) with a SLAx/15-7 MHz transducer (linear type) and a 20-gauge needle with a 0.38-mm J-tip guide-wire (CV Legaforce EX^®^; Terumo Co., Tokyo, Japan). Before starting the punctures, an intensivist supervisor, who specialized in ultrasound-guided central venous catheterization and was double certified by the Japanese Society of Anesthesiologists and the Japanese Society of Intensive Care Medicine, provided a 30-min tutorial to explain and demonstrate the procedure; a video was also presented. After the tutorial, the resident physicians performed the procedures while the supervisor observed the physicians without intervening.

For the SA approach, the transducer was positioned at the puncture site on the manikin to visualize the target vein in the SA view. A needle was inserted close to the transducer at a 30°–45° puncture angle, while visualizing its tip, as it approached the midline axis of the target vein. The needle insertion was repeated if necessary to ensure correct directionality. The needle was then advanced until it penetrated the anterior wall of the target vein. After confirming the initial return of water via the attached syringe, the needle was advanced slightly further at a reduced angle to ensure proper catheter insertion into the target vein, while visualizing the needle tip in the SA view. After removing the inner stylet and reconfirming the location of the cannula by the return of water through the syringe, a guide-wire was inserted.

**Fig 2:**
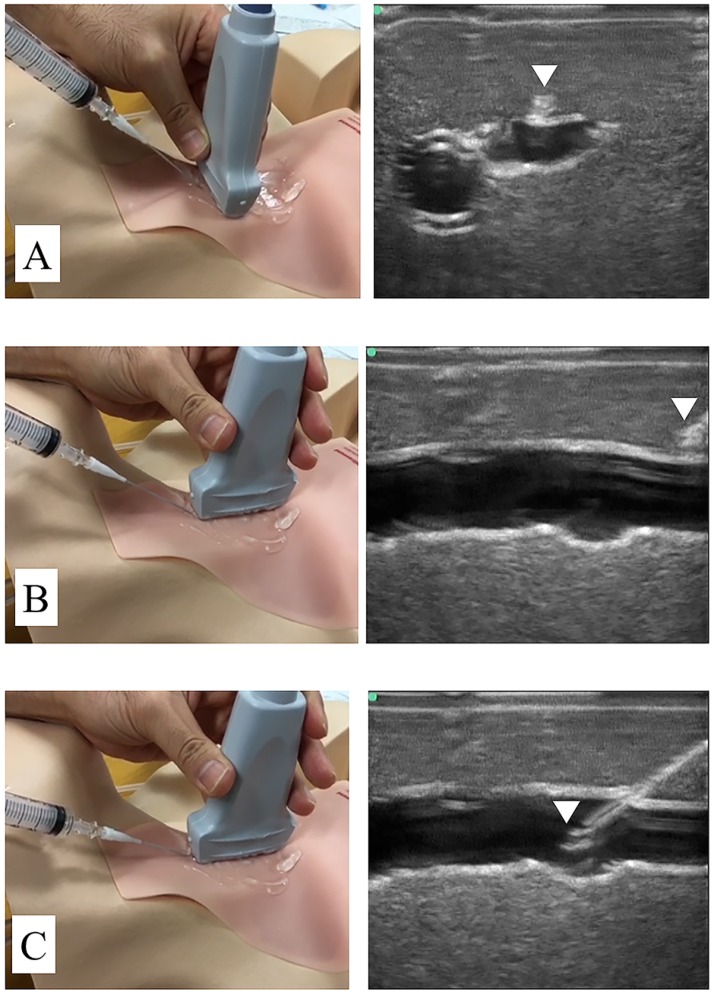
The needle as visualized on ultrasonography. A) The needle tip (arrow) is visualized as a dot between the skin and anterior wall of the target vein in the short-axis view. B) After rotating the transducer by 90°, the entire length of the needle (arrow) is observed in the long-axis view. C) The needle (arrow) is observed puncturing the anterior wall of the target vein.

For the SLA approach, the transducer was initially positioned at the puncture site on the manikin to visualize the target vein in the SA view. A needle was inserted close to the transducer at a 30°–45° puncture angle, while visualizing the needle tip as it approached the midline axis of the target vein (Fig 2A). The needle insertion was repeated if necessary to ensure the correct angle. The transducer was then rotated by 90° while maintaining the needle *in situ* to visualize its entire length in the LA view (Fig 2B). The needle was subsequently advanced through the anterior wall of the target vein while under continuous observation (Fig 2C). After confirming the initial return of water via the connected syringe, the needle was advanced slightly further at a reduced angle to allow for proper catheter insertion into the target vein, while attempting to maintain visualization of the needle tip in the LA view. After removing the inner stylet and reconfirming the location of the cannula by the return of water through the syringe, a guide-wire was inserted.

**Fig 3:**
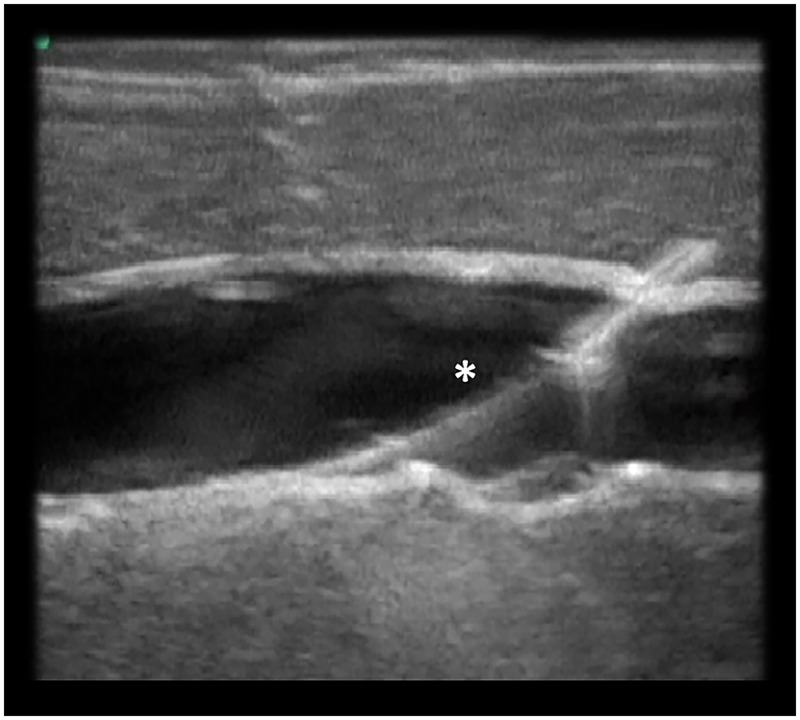
The guide-wire as visualized on ultrasonography. The guide-wire (*) is observed in the long-axis view.

If water was not returned via the cannula, the catheter was withdrawn with aspirating water until the return of water was confirmed through the connected syringe, and the guide-wire was then inserted; in this case, PWP was assumed to have occurred. If the return of the water was not confirmed despite withdrawing the cannula to the surface of the manikin, the needle was assumed not to have punctured the target vein, and puncturing was reattempted using the same procedure, until the guide-wire was successfully inserted into the target vein. The successful positioning of the guide-wire was confirmed by the supervisor using ultrasonography (Fig 3) [21].

### Measurements

We recorded the success of guide-wire insertion, PWP occurrence, needle tip visualization during anterior vessel wall puncture, the duration of the procedure, the number of skin punctures for needle redirection, and the number of attempts during each procedure.

### Study endpoints

The primary endpoint was the rate of procedural success, which was defined as successful guide-wire insertion without the occurrence of PWP and with the needle tip visualized at the time of the anterior wall puncture. The secondary endpoint was the duration of the procedure, which was calculated from the time of initial puncture to the time of guide-wire entry, which was confirmed by the supervisor.

### Statistical analysis

A previous study demonstrated that the rate of needle tip visualization during vessel puncture was 62% and 23% for LA and SA approaches, respectively [22]. We hypothesized that needle tip visualization may be associated with PWP occurrence, and that preventing PWP could guarantee high procedural success rates, which we used as our composite endpoint. No previous studies have evaluated the rate of needle tip visualization during SLA; therefore, we used the rate of LA instead of SLA. Assuming that needle tip visualization using the SLA approach is similar to that with the LA approach, and a correlation between paired observations is equal to 0.40, we estimated that 19 trials would provide 80% power at an α-level of 0.05. The sample size was calculated using Stata Statistical Software version 13.1 (StataCorp, College Station, TX, USA).

Statistical analyses were performed with StatFlex version 6.0 (Artech Co., Ltd., Osaka, Japan) and JMP version 10.0.0 (SAS Institute, Cary, NC, USA) software packages. We used the McNemar test to compare the procedural success rates of the two approaches. The procedure durations were compared using the Wilcoxon signed-rank test. Values are expressed as median [interquartile range]. The number of skin punctures for needle redirection and the number of attempts were compared using paired t-tests. Values are expressed as mean ± SD. A P-value of < 0.05 was considered statistically significant.

## Results

Guide wire insertion was performed successfully in all SA and SLA procedures. Four and nine resident physicians in group A successfully performed the primary endpoint using the SA and SLA procedures, respectively; two and nine in group B achieved the same, respectively (Fig 1). In SA, one resident physician (5%) experienced PWP only, eight (40%) did not visualize the needle tip without PWP, and five (25%) experienced PWP without needle tip visualization, resulting in six (30%) successful procedures. In SLA, two resident physicians (10%) experienced PWP without needle tip visualization, resulting in 18 (90%) successful procedures. Overall, six (30%) and 18 (90%) physicians successfully performed the primary endpoint using the SA and SLA techniques, respectively. Twelve (60%) physicians successfully performed the primary endpoint using the SLA procedure but failed to perform using the SA procedure. None of the resident physicians who successfully performed the primary endpoint using the SA technique failed to complete the SLA technique, while two (10%) failed to perform the primary endpoint in both procedures (Table 1). According to the McNemar test, the SLA approach had a significantly higher procedural success rate than the SA approach (P < 0.001).

**Table 1:** 

		SA approach	
		Procedural success (n = 6)	Procedural failure (n = 14)
SLA approach	Procedural success (n = 18)	6	12
	Procedural failure (n = 2)	0	2

SA, short-axis; SLA, combined short- and long-axis.

The durations of the SLA and SA approaches were 59.5 [46.0–88.5] and 45.0 [37.5–84.0] seconds, respectively. The difference of the duration between the two procedures was 15.5 [0–28.5] seconds. There was no significant difference in duration between the two approaches (P = 0.12). The number of skin punctures for needle redirection were 1.6 ± 0.88 and 1.7 ± 0.73 in SLA and SA, respectively (mean ± SD). There was no significant difference between the two approaches (P = 0.37). The number of attempts were 1.05 ± 0.22 and 1.05 ± 0.22 in SLA and SA, respectively (mean ± SD). There was no significant difference between the two approaches (P = 1.00).

## Discussion

In this study, we found that the SLA approach had a significantly higher procedural success rate than the SA approach when performed by novice operators, with no increased procedural duration. Although this technique has been reported previously [20], the present study demonstrates the superiority of a novel technique that combines the SA and LA approaches.

Using the SLA approach, the needle tip can be initially visualized between the skin and anterior wall of the target vein in the SA view, and the relationship between the needle and adjacent vessels can be easily discerned. Next, the operator switched to the LA view, where needle penetration through the anterior wall of the target vein can be confirmed in real-time, which is the advantage of this approach. It is possible to visualize the needle penetrating the anterior wall of the target vein in real-time using only the LA approach; however, in this approach, the thin ultrasound beam must be aligned with the midline axis of the longitudinal plane of the target vein and with the whole length of the needle, which is quite challenging for novice operators.

Needle tip visualization rate at the time of anterior wall puncture has been reported to be 62% and 23% in LA and SA, respectively [22]. However, in our study, the visualization rate was 90% using the SLA approach, which was significantly higher. In the SLA approach, the direction of the advancing needle and the midline axis of the target vein can be aligned in the SA view, and then, by rotating the probe and overlapping the thin ultrasound beam, the three axes can be aligned and needle tip visualization is improved. When attempting to visualize the entire length of the needle, puncturing the needle precisely in the fixed LA view is quite difficult; however, adjusting the transducer to the fixed needle is comparatively easier.

In this study, resident physicians were able to puncture the target vein with a PWP rate of 10% using the SLA approach. PWP has been reported to occur in 31% and 37% of procedures using the SA and LA approaches, respectively [12]. In another study, researchers have shown that 64% of resident physicians experienced PWP using the SA approach [13]. These results indicate that many operators lose sight of the tip of the needle when performing either the SA or LA technique. However, we overcame this problem by using the SLA approach, which facilitates continued visualization of the needle tip in real-time during the entire procedure.

A recent RCT reported that the success rate of ultrasound-guided central venous catheterization in the subclavian vein was significantly higher when using SA than when using LA [23], which seems contradictory to our results. We speculate that the differences could be due to methodology: the procedure was performed 1) in real patients and 2) not by a novice operator. These methodological differences may account for the differences in experimental outcomes. Moreover, this study endpoint did not include PWP; therefore, the outcomes cannot be compared.

It has been reported that the SA approach is significantly faster than the LA approach when performed by novice physicians [24]; however, the difference in the duration of the two procedures remained unclear. Although the duration of the procedures did not differ significantly between the two techniques in our study, the time required for rotating the transducer and adjusting it to visualize the entire length of the needle in the LA view may have contributed the longer duration of the SLA technique. Nevertheless, a median time difference of approximately 15 seconds would presumably not affect patient outcomes in clinical practice.

### Limitations

This study has several limitations. First, the sample size of this study is small, and we used the rate of needle tip visualization using LA, instead of SLA, and SA approach in sample size calculation. This might have resulted in an underestimation of the required sample size and could be associated with statistical bias. Second, this study was limited to using a manikin, rather than a human body, which shows significant variations in anatomy and collapsible vessels. Further analysis in live patients would be needed. Third, this study involved resident physicians who are not familiar with ultrasound-guided central venous catheterization. Therefore, it is unknown how well-trained physicians will perform the new SLA procedure. Finally, we randomized the order in which the residents performed both approaches. Each resident may gain additional experience by mastering one technique first before switching to the other.

### Conclusions

This study showed that the SLA approach is an easily learned, safer procedure for novice operators performing ultrasound-guided central venous catheterization in a manikin model. The clinical utility of this technique in real patients is worth exploring.
